# Assessing the Effects of β-Triketone Herbicides on the Soil Bacterial and *hppd* Communities: A Lab-to-Field Experiment

**DOI:** 10.3389/fmicb.2020.610298

**Published:** 2021-01-11

**Authors:** Clémence Thiour-Mauprivez, Marion Devers-Lamrani, David Bru, Jérémie Béguet, Aymé Spor, Arnaud Mounier, Lionel Alletto, Christophe Calvayrac, Lise Barthelmebs, Fabrice Martin-Laurent

**Affiliations:** ^1^Biocapteurs-Analyses-Environnement, Universite de Perpignan Via Domitia, Perpignan, France; ^2^Laboratoire de Biodiversité et Biotechnologies Microbiennes, USR 3579 Sorbonne Universités (UPMC) Paris 6 et CNRS Observatoire Océanologique, Banyuls-sur-Mer, France; ^3^Agroécologie, AgroSup Dijon, INRAE, Université Bourgogne Franche-Comté, Dijon, France; ^4^Université de Toulouse, INRAE, UMR AGIR, Castanet-Tolosan, France

**Keywords:** soil, microorganisms, β-triketone, herbicide, *hppd* gene, lab-to-field, non-target organisms

## Abstract

Maize cultivators often use β-triketone herbicides to prevent the growth of weeds in their fields. These herbicides target the 4-HPPD enzyme of dicotyledons. This enzyme, encoded by the *hppd* gene, is widespread among all living organisms including soil bacteria, which are considered as “non-target organisms” by the legislation. Within the framework of the pesticide registration process, the ecotoxicological impact of herbicides on soil microorganisms is solely based on carbon and nitrogen mineralization tests. In this study, we used more extensive approaches to assess with a lab-to-field experiment the risk of β-triketone on the abundance and the diversity of both total and *hppd* soil bacterial communities. Soil microcosms were exposed, under lab conditions, to 1× or 10× the recommended dose of sulcotrione or its commercial product, Decano^®^. Whatever the treatment applied, sulcotrione was fully dissipated from soil after 42 days post-treatment. The abundance and the diversity of both the total and the *hppd* bacterial communities were not affected by the herbicide treatments all along the experiment. Same measurements were led in real agronomical conditions, on three different fields located in the same area cropped with maize: one not exposed to any plant protection products, another one exposed to a series of plant protection products (PPPs) comprising mesotrione, and a last one exposed to different PPPs including mesotrione and tembotrione, two β-triketones. In this latter, the abundance of the *hppd* community varied over time. The diversity of the total and the *hppd* communities evolved over time independently from the treatment received. Only slight but significant transient effects on the abundance of the *hppd* community in one of the tested soil were observed. Our results showed that tested β-triketones have no visible impact toward both total and *hppd* soil bacteria communities.

## Introduction

Soils are known as complex and dynamic habitats where a wide diversity of species lives. Soils could potentially shelter about a quarter of the world biodiversity (Decaëns et al., 2006). Among their inhabitants, microorganisms constitute a huge part of this biodiversity with several billions of bacterial species and more than a hundred thousand fungi species living in one gram of soil (Schloss and Handelsman, 2006). These microorganisms contribute to a series of complex processes and support key soil ecosystem functions associated to food supply, climate regulation or biochemical cycles among others (Wall et al., 2013; Graham et al., 2016). It then appears important to preserve and protect soil microorganisms and their habitats. In 2010, the European Commission officially recognized the need to protect soils and raised them as a non-renewable resource, as this was already the case for water (European Commission, 2010). The European soil biodiversity expert group identified the main pressures on soil organisms and human intensive exploitation appeared as a major threat (Gardi et al., 2013). This intensive exploitation is irremediably linked to the use of plant protection products (PPPs), applied in conventional agriculture to protect crops from various pests. Among them, herbicides are still widely used to control weeds which enter in competition with crops. Some of them, developed during these last two decades are inspired by the biomimetism. They mimicked active natural products and are consequently considered as “eco-friendly” (Cantrell et al., 2012).

This is the case for β-triketone herbicides, for which the chemical structure derived from a natural phytotoxin obtained from the Californian bottlebrush plant, *Callistemon citrinus* (Mitchell et al., 2001). Three β-triketone active substances are mainly used as herbicides on maize cultures: sulcotrione, mesotrione, and tembotrione (Schulz et al., 1993; Mitchell et al., 2001; Lee et al., 2008). These molecules inhibit the 4-hydroxyphenylpyruvate dioxygenase (HPPD) and lead to bleaching and death of weeds (Dayan et al., 2007; Rocaboy-Faquet et al., 2014). This enzyme is not only found in plants but in almost all living organisms, including microorganisms where it takes part to the tyrosine degradation pathway (Moran, 2005). The *hppd* gene, coding the targeted enzyme, is described in about 2000 bacterial species (Thiour-Mauprivez et al., 2020). Thus, soil bacteria, classified as “non-target organisms” by current EU regulation for pesticide authorization, might be impacted by β-triketones, with a possible domino effect on microbial functions supporting soil ecosystem services (Thiour-Mauprivez et al., 2019). In this context, studies assessing the ecotoxicological impact of β-triketone herbicides on soil total bacteria have recently been led (Romdhane et al., 2016a, 2019). In order to get a closer look to the direct effect of β-triketone, we report here the assessment of its ecotoxicological effect on the abundance and the diversity of the total bacterial community and the bacterial community harboring the *hppd* gene. These parameters were measured in a lab-to-field experiment in which lab conditions were led with the active substance at higher doses than the recommended ones, to test the “worst-case scenario” and field experiments were led with recommended field doses (RfDs), in frame with a “realistic scenario of exposure,” as recommended by the European Food Safety Authority (Ockleford et al., 2017). Our lab-to-field experiment complies the two-tiered approach to assess the possible toxicity of β-triketones on the *hppd* bacterial community which harbors the gene encoding the enzyme targeted by these herbicides.

Under lab conditions, soil microcosms were treated with 1× or 10× the RfD of sulcotrione using the active ingredient or the formulated product (Decano^®^) or not treated (control). Under these conditions, dissipation studies of the active substance or the formulated PPP in soil microcosms were carried out in order to estimate the scenario of exposure of soil microorganisms. Under field conditions, samples were collected in maize crops exposed to PPP treatments comprising several β-triketones or not treated at all (control). For both lab and field experiments, the abundance and the diversity of both the total and the *hppd* bacterial communities were estimated by quantitative PCR (qPCR) and high throughput sequencing, respectively.

## Materials and Methods

### Laboratory Experiment

#### Reagents

Standard of sulcotrione [(S) 98.8% purity], a weak acidic herbicide with pKa value of 2.87 and MW = 328.7 g.mol^–1^ was purchased from Sigma-Aldrich, France. Formulated sulcotrione [Decano^®^ (D)] was purchased from (SAPEC Agro, France). Methanol and acetonitrile (HPLC quality) were purchased from Carlo Erba, dichloromethane for pesticide analysis from Riedel-de-Haën GmbH, trifluoroacetic acid (99.0% purity) and hydrochloric acid (38.0%) from Aldrich and water was Milli-Q quality.

#### Soil Sampling and Microcosm Set-up Under Lab Conditions

Soil samples were collected from the surface layer (0–20 cm) of an arable field located in Perpignan, France. According to the World Reference Base of Soil Resources, it is a sandy loam clay-rich soil composed of 16.2% clay, 29.1% silt, and 54.7% sand. Granulometry was measured according to the particle size 5 fractions with no decarbonization method (NF X 31-107). Soil contains 27.5 g/kg of organic matter, 15.99 g/kg of organic carbon, and 1.25 g/kg of nitrogen. These contents were measured by dry combustion according to the standard methods NF ISO 10694 and NF ISO 13878. Soil physicochemical characteristics are 99 meq/kg Cation Exchange Capacity (CEC), 260.3% Ca2/CEC and water pH 8.04. CEC was measured according to the Metson method (NF X 31-130) and water pH was determined using a glass electrode (NF ISO 10390). Soil samples were sieved to 2 mm and soil moisture was of 3%. Soil samples were divided in microcosm of 20 g of soil each and treated or not with active ingredient {2-[2-chloro-4-(methylsulfonyl)benzoyl]-1,3-cyclohexanedione} (S) or formulated compound [Decano^®^ (D)]. For each treatment (sulcotrione or Decano^®^), twelve microcosms were treated at 1× RfD (1.5 μg/g), twelve were treated at 10× RfD (15 μg/g) and twelve remained untreated (control). These doses were chosen to fulfill the two-tier scenario of exposure. Soil moisture was adjusted at 22% relative humidity. Soil microcosm were incubated at 21 ± 2°C in the dark. Every 2 days, microcosms were ventilated and soil moisture was adjusted if needed under sterile conditions. Soil samples were collected after 30 min of treatment (day 0), 7, 14, and 42 days of incubation and immediately stored at −20°C for further analyses. Three replicates were prepared for each treatment (S control, S 1× RfD, S 10× RfD, D control, D 1× RfD, and D 10× RfD).

#### Herbicide Quantification

##### Extraction step

This analytical method was an adaptation of the procedure previously published by Chaabane et al., 2008. In order to follow active substance or formulated PPP dissipation in soil, 10 g of soil (triplicates) for each condition were extracted twice with 30 mL of acetonitrile/0.1 M hydrochloric acid (90/10; V/V) under agitation at 100 rpm orbital shaking for 50 min and then filtered on a Whatman filter GF/A 47 mm. The organic filtrate was evaporated at 30°C and the acidic aqueous solution was then extracted twice with 6.0 mL of dichloromethane. The organic phase was evaporated to dryness and then solubilized with 3.0 mL of methanol. The final extract was analyzed by High Performance Liquid Chromatography (HPLC).

##### Chromatographic analysis

Soil extracts treated with sulcotrione were analyzed using a VWR Hitachi LaChrom apparatus consisted of auto-sampler L-2200 and HTA L-2130 pump modules equipped with a Kinetex C18 column (5 μm, 150 mm × 3 mm) and a DAD L-2450 UV/Vis detector set at 285 nm. For sulcotrione, the mobile phase consisted of a mixture of water acidified with 0.1% acetic acid (AW) and acetonitrile (ACN) delivered at a flow-rate of 0.5 mL/min with an isocratic mode 70/30 (AW/ACN) for 15 min. For soil extracts treated with Decano^®^, the mobile phase was the same as the one used for sulcotrione but was delivered at a flow-rate of 1 mL/min with an isocratic mode 80/20 (AW/ACN) for 8 min.

##### Analytical performance

Recovered analytics were evaluated by spiking soil samples replicates (*n* = 5) with increasing doses (0.1–75 μg/g) of analytical standards of sulcotrione or formulated sulcotrione (Decano^®^) prior to extraction. The mean recovery rates were estimated as 85 ± 10 and 52 ± 4%, for sulcotrione and Decano^®^, respectively. The limit of quantification, defined as the sample concentration required to give a signal-to-noise ratio of 5:1 was evaluated to 0.1 μg/g for both sulcotrione and Decano^®^.

### Field Experiment

Soil samples were collected from the surface layer (0–20 cm) of three arable fields located near Tarbes, France. These fields were not part of an experimental parcel, they were exploited by farmers and, thus, were treated at RfDs of PPPs with a crop protection program which we did not choose. Field 1 has not received any PPPs for 3 years and was historically cultivated with maize but is now cultivated with walnut tree and will constitute the control field. Field 2 was sown with maize on the 30th April 2019 and was treated with Camix^®^ (containing 40 g/L mesotrione, applied at 2.5 L/ha) on the 23th May 2019 and with Auxo^®^ (containing 50 g/L tembotrione, applied at 0.6 L/ha), Nisshin (nicosulfuron-based, 0.6 L/ha), Banvel (dicamba-based, 0.2 L/ha), and Sakol (alkylpolyglucoside-based, 0.1 L/ha) on the 3rd June 2019. Three samplings were made over one crop cycle on these three fields with three replicates made of seven composite-samples each done using an auger. The first sampling time was before seedling and treatments on the 16th September 2018 (September 2018). The second was made after treatment for field 3 whereas in the field 2, it was made between the first and the second treatment on the 6th June 2019 (June 2019). For all the fields, the last sampling campaign was led on the 28th August 2019 (August 2019). Soil properties and chemical characteristics are described in the Supplementary Table 1. Soil samples were stored at −20°C until use.

### Ecotoxicological Impact of β-Triketone on Soil Bacterial Communities

#### Soil DNA Extraction

At each sampling time of the experiment, nucleic acids were extracted with RNeasy PowerSoil DNA Elution Kit (Qiagen). DNA quality was checked and concentrations quantified by Quant-iT^TM^ PicoGreen^TM^ (Thermo Fischer Scientific, France). A mean of 0.19 ± 0.18 and 0.38 ± 0.29 μg/g of soil were extracted from laboratory and field samples, respectively. DNA extracts were stored at −20°C until use.

#### Abundance of Total and *hppd* Bacterial Communities

Prior to quantification, inhibition tests were performed as previously described in Petric et al. (2011), in order to detect the presence of DNA co-extracts possibly inhibiting the qPCR assays. Samples were then diluted at 1 ng/μl. Amplification of both *hppd* and 16S rRNA genes were carried out using a ViiA^TM^ 7 (Thermo Fisher Scientific, France) in a final reaction mixture of 15 μL. The mixture contained SYBR Green PCR Master Mix (Takyon^TM^ Low ROX SYBR^®^ 2× Mastermix Blue, Eurogentec, Belgium), 250 ng of T4 gp32 (Qbiogene, MP Biomedicals, France), 10 μM of each *hppd* primer (HIAF and FFER, Thiour-Mauprivez et al., 2020), or 1 μM of each 16S rRNA primer (341F and 534R, Muyzer et al., 1996) and 1 ng of soil DNA template. qPCR runs were as follow for the amplification of the *hppd* gene: *Taq* polymerase enzyme activation step for 3 min at 95°C, then 40 cycles of 10 s of denaturation at 95°C, 30 s of annealing at 54°C, elongation for 30 s at 72°C, and a melting curve stage with 15 s at 95°C, 1 min at 68°C, and 15 s at 95°C (data were collected at this step). Run parameters used for the amplification of 16S rRNA gene were previously described by Romdhane et al. (2016a). Standard curves were generated using serial dilutions of linearized plasmid pGEM-T easy vector system containing each standard gene sequence (ranging from 10^2^ to 10^7^ copies per qPCR reaction). For each condition tested, two qPCR assays were conducted. In each assay, qPCR calibration was performed in triplicate and three no-template controls (NTC) were also included.

#### Diversity of Total and *hppd* Bacterial Communities

Total and *hppd* bacterial diversities were monitored using a high throughput sequencing of 16S rRNA and *hppd* amplicons, respectively (Illumina MiSeq, Microsynth). A two-step PCR procedure was used to amplify the *hppd* gene from obtained DNA extracts. In the first step, 27 cycles of amplification were performed in duplicate using the NGS_FFER-NGS_HIAF primer pair (Thiour-Mauprivez et al., 2020). Duplicates were then pooled and a second step of PCR was conducted, in duplicate, using 1 μL of the previous PCR as template to carry out eight cycles of amplification with barcoded primers. To amplify the 16S rRNA gene from obtained DNA extracts, 20 cycles of amplification were performed in duplicate using the U341F-805R primer pair (Takahashi et al., 2014). Duplicates were then pooled and a second step of PCR was conducted, in duplicate, using 1 μL of the previous PCR as template to carry out 15 cycles of amplification with barcoded primers. After pooling, amplicon size was checked by electrophoresis on a 2% agarose gel. 25 μL of PCR products were normalized using SequalPrep^TM^ Normalization Plate Kit (Invitrogen) and 10 μL of each sample were sent to Microsynth for sequencing. 16S rRNA amplicon data analysis were conducted as described in Romdhane et al. (2016a). For the *hppd* amplicon data analysis, reads were quality-controlled and assembled using PEAR (Zhang et al., 2014). Unassembled reads and once-assembled reads outside the expected range were discarded. Sequences were corrected and converted into protein sequences using framebot (Wang et al., 2013), then clustered into OTUs using cd-hit (Li and Godzik, 2006). Representative *hppd* sequences for each OTU were aligned using Muscle (Edgar, 2004) and the maximum likelihood phylogeny was calculated using FastTree 2 algorithm (Price et al., 2009). Several α-diversity indices (PD whole tree, Simpson reciprocal, and Shannon indices) were then calculated using rarefied OTU tables at the depth of 6000 sequences per sample in the microcosms treated with sulcotrione, 15,000 sequences per sample in those treated with Decano^®^ and 20,000 sequences per sample in the soils sampled directly from fields considering the 16S rRNA amplicons. Same indices were calculated using rarefied OTU tables of 2300 sequences per sample in the microcosms treated with sulcotrione, 2000 sequences per sample in those treated with Decano^®^ and 4200 sequences per sample in the soils sampled directly from fields considering the *hppd* amplicons. Principal Coordinate Analysis (PCoA) plots were generated in QIIME based on weighted UniFrac distance matrix and coordinates were used to draw 3D figures.

### Statistical Analysis

Statistical analysis were conducted using R software and a significance threshold was set at a *p*-value of 0.05 (R Development Core Team, 2005). For qPCR results, data were log-transformed. Data normality was verified by a Shapiro–Wilk test. The homogeneity of variances of the residues was verified by a Levene test. Data were subjected to an analysis of variance (ANOVA) and Tukey test was performed for each treatment and each time point. Not all data showed a normal distribution. Consequently, non-parametric test (Kruskal–Wallis test) was carried out on non-normal distributed data. Weighted or unweighted UniFrac distance matrices were subjected to a PERMANOVA analysis using the ADONIS function from R package “vegan” (Oksanen et al., 2020). Sparse Partial Least Squares Discriminant Analysis (sPLS-DA) was performed to select discriminant OTUs between different treatments using the function “splsda” from R package mixOmics (Rohart et al., 2017). A homemade R script was used to identify the discriminant OTUs by performing an ANOVA on each of these OTUs.

## Results

### Fate of Sulcotrione and Decano^®^ in Soil Microcosms

In order to decipher the exposure scenario of bacteria in soil microcosms, kinetics of dissipation of sulcotrione and Decano^®^ in soils were measured at each time point (Table 1). Sulcotrione and Decano^®^ dissipation kinetics could be reasonably described by a first-order kinetics (C = C0.e^–*kt*^) for the application at 1× RfD. Half-life time of sulcotrione in Perpignan soil was estimated at *ca* 4 days for 1× RfD. Regarding Decano^®^, half-life time in Perpignan soil was evaluated at *ca* 1.5 days for the 1× RfD condition. For the application at 10× RfD, we were only able to estimate graphically the half-life time of sulcotrione at *ca* 14 days and of Decano^®^ at *ca* 18 days (Supplementary Figure 1). Anyhow, sulcotrione and Decano^®^ both reached an unquantifiable concentration after 42 days of exposure in Perpignan soil, whatever the starting concentration of RfD applied.

**TABLE 1:** Main environmental parameters of sulcotrione and Decano^®^ estimated by modeling their kinetics of dissipation in Perpignan soil.

**Treatment**	**Sulcotrione**		**Decano^®^**	
RfD	1	10	1	10
k (day)	0.36 ± 0.02	n.d.	0.43 ± 0.03	n.d.
DT_50_ (day)	1.94 ± 0.08	n.d.	1.63 ± 0.11	n.d.
*R*^2^	0.81 ± 0.10	n.d.	0.99 ± 0.01	n.d.

### Impact of Sulcotrione and Decano^®^ on the Abundance of the Total and the *hppd* Bacterial Community

**FIGURE 1:**
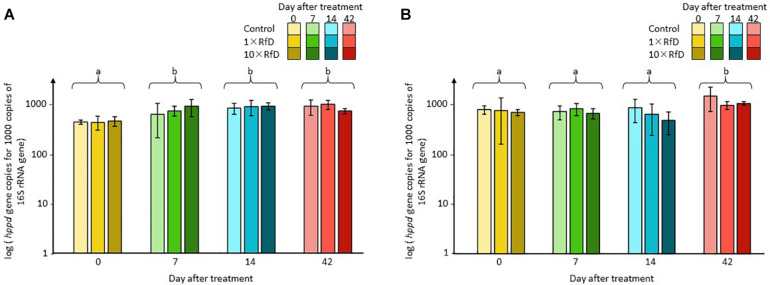
*hppd* sequence copy number per 1000 sequences of 16S rRNA. Soil microcosms **(A)** treated with sulcotrione; **(B)** with Decano^®^. Values shown are averages of *n* = 3 biological replicates for each series. Error bars are calculated from the standard deviation for each series. ANOVA and Kruskal–Wallis test *p* < 0.05 were considered as significant and, if needed, indicated by a small letter.

The impact of sulcotrione and Decano^®^ on the abundance of the total and the *hppd* bacterial community in the soil microcosms was monitored by qPCR. Sequence copy numbers ranged from 8.99 × 10^3^ to 3.82 × 10^5^ sequences of 16S rRNA per nanogram of DNA and from 4.81 × 10^3^ to 2.75 × 10^5^ sequences of *hppd* per nanogram of DNA. The abundances of both the total and the *hppd* bacterial communities were not affected by the β-triketone treatments applied. Similarly, the relative abundance of *hppd* sequences per 1000 sequences of 16S rRNA (Figure 1) was not affected by any of the β-triketone treatments applied. In both cases, only a time effect was observable with relative abundance measured at day 0 different from the other sampling days (ANOVA, *p* = 0.001) for microcosms treated with sulcotrione and relative abundance measured at day 42 different from those measured at 0, 7, or 14 days (Kruskal–Wallis test, *p* = 0.002) for microcosms treated with Decano^®^.

### Impact of Sulcotrione and Decano^®^ on the Diversity of the *hppd* Bacterial Community

To further investigate the possible ecotoxicological impact of sulcotrione and Decano^®^ on the diversity of total and *hppd* bacterial communities, the 16S rRNA and the *hppd* amplicons were amplified from soil DNA extracts and sequenced. These sequences were grouped into 2849 OTUs and 1688 OTUs at 80% amino-acid sequence identity threshold for sulcotrione and Decano^®^ treatment respectively. α-Diversity was estimated for each treatment by a range of indices depicted in Table 2. No significant differences neither overtime nor in response to the RfD applied was shown for all tested treatments. Same measurements were made on the total bacterial α-diversity by sequencing the 16S rRNA gene. Sequences were grouped in 4099 OTUs in the soil treated with sulcotrione and in 4767 OTUs in the soil treated with Decano^®^ at 94% nucleotide sequence identity threshold. A time effect was highlighted but no significant differences due to the treatment applied were shown (Supplementary Table 2). Together, these results indicate that the α-diversity of both the total and the *hppd* communities are not affected by sulcotrione or Decano^®^ in our experimental conditions.

**TABLE 2:** Richness and diversity indices of the *hppd* bacterial community calculated for soil microcosms exposed for 0, 7, 14, and 42 days to sulcotrione (S, in brown) or Decano^®^ (D, in blue) applied at different concentrations (control, 1× RfD, and 10× RfD).

**Day after treatment**	**Treatment**	**Observed species**	**PD whole tree**	**Simpson reciprocal**
0	S control	21124	625	186
	S 1× RfD	18826	557	162
	S 10× RfD	18449	5413	158
	D control	19617	524	162
	D 1× RfD	21638	598	2617
	D 10× RfD	20015	542	171
7	S control	16521	496	92
	S 1× RfD	19228	566	166
	S 10× RfD	16711	493	111
	D control	20624	556	217
	D 1× RfD	20817	574	2614
	D 10× RfD	23312	631	261
14	S control	17728	507	111
	S 1× RfD	16818	485	91
	S 10× RfD	18035	527	102
	D control	2043	552	181
	D 1× RfD	23024	615	2611
	D 10× RfD	22517	595	255
42	S control	17621.2	515	112
	S 1× RfD	16725	506	122
	S 10× RfD	19931	578	144
	D control	2111	570	191
	D 1× RfD	2137	571	192
	D 10× RfD	20411	573	162

*Mean values ± confidence intervals are shown. No significant difference for each treatment and each time point was shown (Kruskal–Wallis test *p* < 0.05 were considered as significant and, if needed, indicated by a small letter).*

**FIGURE 2:**
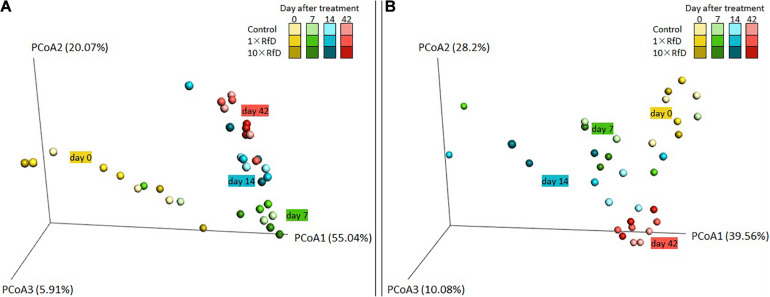
UniFrac analysis of the effect of sulcotrione **(A)** and Decano^®^ **(B)** applied at different concentrations (control, 1× RfD, and 10× RfD) on the *hppd* bacterial community composition of soil microcosms at 0, 7, 14, and 42 days. The first three axes of the PCoA of the weighted UniFrac distance matrix of *hppd* amplicon Illumina sequencing are shown. The percent of variance explained by each axis is given.

The ecotoxicological impact of sulcotrione and Decano^®^ on the *hppd* bacterial β-diversity of soil microcosms was evaluated. For each treatment, the analysis of the PCoA, representing the weighted UniFrac distances, showed no significant difference in the *hppd* bacterial community composition between RfD applied (Control, 1× RfD, or 10× RfD) and sampling time (0, 7, 14, 42 days after treatment) (Figure 2).

Altogether, the results obtained for α- and β-diversity measurements indicate that the diversity of the *hppd* bacterial community was not modified over time or according to the RfD applied. Same conclusions were made on the measurements of the total bacterial diversity, monitored by 16S rRNA Illumina sequencing (Supplementary Figure 2).

### Abundance and Diversity of the Total and the *hppd* Bacterial Community in Agricultural Soils Exposed to β-Triketone Herbicides

The ecotoxicological impact of PPP treatments on the abundance, the diversity and the composition of the total and the *hppd* bacterial communities was also assessed under real agronomical conditions.

**FIGURE 3:**
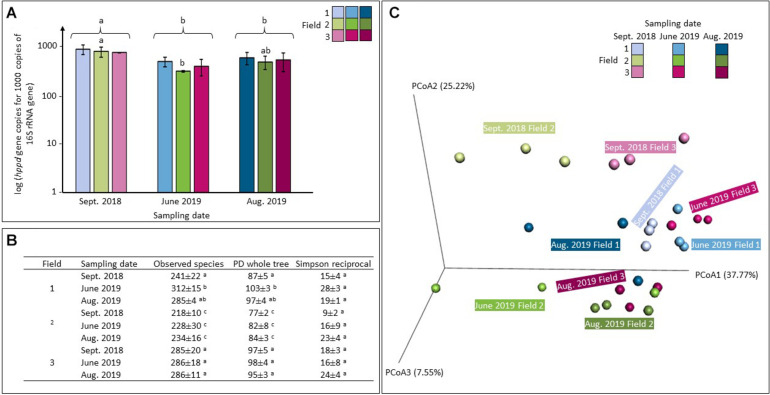
Analysis of the abundance **(A)**, the α-diversity **(B)**, and the β-diversity **(C)** of the *hppd* bacterial community in soils of agricultural exposed to PPPs (fields 2 and 3) or not (field 1) collected at different sampling times over a 1-year crop cycle (September 2018, June 2019, August 2019). **(A)** *hppd* sequence copy number per 1000 sequences of 16S rRNA gene. Values shown are averages of *n* = 3 biological replicates for each series. Error bars are calculated from the standard deviation for each series. ANOVA *p* < 0.05 was considered as significant and, if needed, indicated by a small letter. **(B)** Richness and diversity indices of the *hppd* bacterial community (mean values ± confidence intervals). ANOVA tests were conducted and *p* < 0.05 was considered as significant and indicated by a different letter. **(C)** UniFrac analysis on the *hppd* bacterial community composition.

Sequence copy numbers ranged from 1.21 × 10^5^ to 4.63 × 10^5^ sequences of 16S rRNA per nanograms of DNA and from 7.90 × 10^4^ to 2.62 × 10^5^ sequences of *hppd* per ng of DNA. The relative abundance of *hppd* gene copies per 1000 sequences of 16S rRNA is shown in Figure 3A. An ANOVA test suggest that whatever the treatment applied, values recorded in the samples collected in September 2018 were significantly higher than that of the two others (June 2019 and August 2019) (*p* < 0.01). Considering the relative abundance of *hppd* found in each treated soil, only values observed in field 2 were statistically different from those measured in the others: values recorded in June 2019 (ANOVA, *p* < 0.01) were significantly lower than those of September 2018. This was no longer observed on samples of August 2019, which were not statistically different from those obtained in September 2018 (ANOVA, *p* = 0.09) or in June 2019 (ANOVA, *p* = 0.17).

The *hppd* sequences were grouped into 1562 OTUs at 80% amino-acid sequence identity threshold. α-Diversity was estimated for each condition by a range of indices depicted in Figure 3B. The observed species and the Phylogenetic Diversity (PD) indices calculated in field 2 were significantly lower than those of field 1 and field 3, no matter what the sampling date (ANOVA, *p* < 0.01). Same measurements were made on the total bacterial α-diversity and they led to the similar conclusions: the three indices calculated in the case of for field 2 were significantly lower from those calculated for fields 1 and 3, respectively (ANOVA, *p* < 0.05, Supplementary Figure 3). The total and the *hppd* bacterial α-diversities are different in field 2 than in the other two fields. On June 2019, the observed species and the PD whole tree indices calculated on the *hppd* bacterial α-diversities within field 1 were significantly different from those calculated on September 2018 (ANOVA, *p* < 0.01) but not from those measured in August 2019 (ANOVA, p_*observed_species*_ = 0.66 and p_*PD_whole_tree*_ = 0.88). It has to be noticed that the α-diversity of the total bacterial community was also modified over time (ANOVA, *p* < 0.05, Supplementary Figure 3).

The *hppd* bacterial β-diversity is illustrated on Figure 3C by a PCoA representing the weighted UniFrac distances. In addition, an sPLS-DA was performed in parallel to an ANOVA test performed on each *hppd* OTU taken separately. All of these tests showed no statistical differences between soils: the β-diversity of *hppd* bacterial community remained unchanged among time and between the different studied soils. Same tests were performed on the total bacterial β-diversity obtained by sequencing the 16S rRNA gene with sequences grouped in 5739 OTUs at 94% nucleotide sequence identity threshold. Whatever the soil considered, no significant differences were found (data not shown).

## Discussion

The aim of this study was to evaluate, in a lab-to-field experimental design, the ecotoxicological impact of β-triketone herbicides on the abundance, the composition and the diversity of total and *hppd* bacterial communities. Under lab conditions, the worst-case scenario (up to 10 times the agronomical dose) as well as the effect of the formulation (sulcotrione vs Decano^®^) were tested. Under field conditions, agronomical scenario exposure including the application of several PPP treatments over a 1-year cropping cycle was tested.

### Estimation of the Fate and Ecotoxicological Impact of Sulcotrione and Decano^®^ on the Total and *hppd* Bacterial Communities Under Lab Conditions

Under lab conditions, soil microcosms were exposed for 42 days to 1× or 10× RfD of sulcotrione or Decano^®^. Kinetics of dissipation of the active substance were established. Although the dissipation of sulcotrione in the Perpignan soil has already been monitored in several studies (Chaabane et al., 2008; Romdhane et al., 2019), this is the first time that the kinetic of dissipation of the active substance of Decano^®^ was measured in this soil. Even if the recovery rate of sulcotrione from microcosms treated with Decano^®^ was not optimal (52%), one could observe that, 42 days after application, sulcotrione was fully dissipated from both batches of microcosms. Our results are consistent with previous studies where similar concentrations were applied, showing a complete dissipation of sulcotrione within 45 days in Perpignan soil (Calvayrac et al., 2012; Romdhane et al., 2016b, 2019). Observed DT_50_ at 1× RfD were in the range of those measured in other studies (4–7 days in dark) confirming that sulcotrione is rapidly dissipated from soils (Barchanska et al., 2016). Concomitantly to sulcotrione dissipation and, as previously observed by Romdhane et al. (2016b), CMBA, the principal transformation product of sulcotrione, is accumulated in Perpignan soil in both microcosm batches. As expected, for both Decano^®^ and sulcotrione, at a higher dose rate of application, dissipation lasted longer than at lower rate of application.

The effect of sulcotrione and of Decano^®^ on the abundance and the diversity of both the total and *hppd* soil bacterial communities was measured by qPCR and amplicon Illumina sequencing, respectively. Both treatments had no effect on the abundance of the total soil bacterial community. This observation is in accordance with that of Romdhane et al. (2016a) showing that natural triketone (i.e., leptospermone) has either no effect or very short-lasting effect on the abundance of the total bacterial community. It has to be noticed that mesotrione, another β-triketone, was shown to transiently modify the abundance and the diversity of both soil bacterial and fungal communities (Du et al., 2018). It suggests that β-triketone ecotoxicological effect on microorganisms depends on the active compound, on the soil type and on their interactions. Similarly, the two treatments, at both doses, had no effect on the composition and structure of the total bacterial community. This is in agreement with Romdhane et al. (2019) who reported no change of α- and β-diversities of the total bacterial community in the soil of Perpignan exposed to either 1× or 10× RfD of sulcotrione. Interestingly, one can observe that different studies carried out on the same soil at different times with different sequencing technologies (454 pyrosequencing vs Illumina sequencing) under similar conditions led to similar conclusions showing the reliability of such approaches, as already suggested (Luo et al., 2012) and consolidating our observations.

Soil bacterial *hppd* abundance and diversity have already been monitored in Perpignan soil (Thiour-Mauprivez et al., 2020) but, to our best knowledge, this was the first time they were monitored in response to a sulcotrione and Decano^®^ exposure. As observed for the total bacterial community, both treatments applied at both doses have no effect on the abundance and diversity of the *hppd* bacterial community. Therefore, one can conclude that, under our experimental conditions, the *hppd* bacterial community harboring the gene target of β-triketone herbicides is insensitive to formulated sulcotrione or to the active substance. The low persistence of sulcotrione in Perpignan soil indicates that soil bacterial community is only shortly exposed to β-triketone and such a short exposure is not enough to modify the abundance or the diversity of the soil bacterial community. In addition, detoxification mechanisms such as efflux transporters, known as unspecific drivers of resistance of bacteria to antibiotics, could work as a detoxification mechanism against β-triketones and even could stop them entering the cell. Such mechanisms have already been observed in an *Escherichia coli* strain exposed to glyphosate (Staub et al., 2012). However, another β-triketone formulated product called Callisto^®^ (mesotrione) is shown to transiently inhibit the nitrification process in bacteria when applied at 10× RfD, probably because of a direct effect (Crouzet et al., 2016). This further reinforces the assumption that like for other herbicidal active substances, the β-triketone ecotoxicological effect on microorganisms depends on the properties of active compound, of soil type and of their interactions.

### Estimation of the Ecotoxicological Effect of β-Triketone on the Abundance, Composition, and Diversity of Both Total- and *hppd*-Bacterial Communities Under Field Conditions

The lab-to-field assessment of the ecotoxicological impact of active substances on soil microorganisms has been proposed as a gold standard to conduct environmental risk assessment of pesticides (Karpouzas et al., 2016). Consequently, in parallel to lab-experiments, the impact of β-triketone PPPs, including mesotrione- and/or tembotrione-based PPPs was measured on the abundance, the composition and the diversity of both the total and the *hppd* bacterial communities in field samples collected over 1 year cropping cycle.

The main result of this field study concerns one of the tested fields (field 2), in which the *hppd* bacterial community abundance decreased after the first treatment with mesotrione-based herbicide (Camix^®^) to finally increase after the second treatment with tembotrione-, dicamba-, nicosulfuron-, and alkylpolyglucoside-based herbicides but without reaching the level of abundance measured initially. This suggests a partial resilience of the *hppd* community in this field. This observed time effect might be due to different factors such as climatic conditions or the phenology of the crop. It could also be explained by the PPP treatments applied to this field. However, we are not able to conclude whether the abundance decrease is due to mesotrione because: (i) Camix^®^ is composed, in addition to mesotrione, of S-metolachlor (400 g/L) and benoxacor (20 g/L); (ii) the α-diversity of this field is significantly lower than those measured in fields 1 and 3, making a comparison uneasy. Regarding the β-diversity measured in this field, it remained unchanged and identical to the other fields all along the cropping cycle.

This field experiment reveals the difficulty to define reliable references when working on formulated product composed of several active compounds with different action modes and of several other compounds entering in their formulation that are usually bound by confidentiality by the agrochemical firms. Considering this, it becomes evident that the conclusions of *a priori* risk assessment carried out under control conditions for a single active compound cannot be expanded for a formulated PPP applied in combination with others in the field all along the cropping cycle. This study also highlights the difficulty to define reliable controls when working on real agronomical situations encountered on farms. Indeed, in our experimental design, we chose a control field not exposed to β-triketones located closely to the other two sampled fields cropped with maize and exposed to herbicides. Even if the soils of chosen fields are subjected to the same pedoclimatic conditions and harbor similar physicochemical properties, differences in the bacterial composition were readily observed at the first sampling date. This raises the question of the reference to interpret the changes in response to the PPP treatments applied. Consequently, there is a need to define a normal operating range (NOR) for each studied indicator as it has been evaluated on diverse indicators (soil microbial biomass and bacterial diversity) in a 13 km^2^ parcel on 278 soils all spaced from 215 meters (Constancias et al., 2015). The fully randomized block design that is usually applied in agronomical assay has been shown in the past to be of interest to estimate the impact of several active substances applied one by one on soil microorganisms (Papadopoulou et al., 2016; Storck et al., 2018), but it seems not appropriate to evaluate the risk encountered in-farm at a systemic level which is far more complex with a combination of PPPs applied simultaneously or one after the other all along the cropping cycle.

## Conclusion

Our study provides a comprehensive lab-to-field assessment of the ecotoxicity of β-triketone herbicides (active compound and formulated one) on both the total and *hppd* bacterial communities. Our lab-experiment confirmed the low persistence of the active substance in both sulcotrione and Decano^®^ treated soil microcosms. Under our experimental conditions, both treatments at agronomical doses have no effect on the abundance, the composition and the diversity of both total and *hppd* bacterial communities. Our field-experiment led to similar conclusions thereby comforting the eco-friendly reputation of β-triketone herbicides.

## Data Availability Statement

The datasets presented in this study can be found in online repositories. The names of the repository/repositories and accession number(s) can be found below: NCBI, accession number: PRJNA666084 (https://www.ncbi.nlm.nih.gov/sra/?term=PRJNA666084).

## Author Contributions

CT-M, LB, FM-L, MD-L, and CC conceived and designed the research. CT-M and CC conducted the chemical analysis. CT-M, MD-L, AS, and AM conducted the bioinformatics analysis. JB and DB conducted the PCR experiments. LA conducted the field sampling. CT-M, FM-L, and LB wrote the manuscript. All authors read and approved the manuscript.

## Conflict of Interest

The authors declare that the research was conducted in the absence of any commercial or financial relationships that could be construed as a potential conflict of interest.
