# Supplementary material for: Assessing the Effects of β-Triketone Herbicides on the Soil Bacterial and hppd Communities: A Lab-to-Field Experiment
Source: Front Microbiol. 2021 Jan 11;11:610298. doi: 10.3389/fmicb.2020.610298 (PMC7829504; doi:10.3389/fmicb.2020.610298)
Supplement: Supplementary file 1 [file Data_Sheet_1.docx]

Table S1. Soil properties of samples collected in three agricultural fields located in the same area. Granulometry was measured according to the particle size 5 fractions with no decarbonisation method (NF X 31-107) and revealed sandy clay loam soils. Organic carbon, matter and nitrogen contents were measured by dry combustion according to the standard methods NF ISO 10694 and NF ISO 13878. CEC was measured according to the Metson method (NF X 31-130). n.d=not determined.

| Field | Sampling date | Clay (%) | Silt (%) | Sand (%) | Water pH | Organic carbon (g/kg) | Organic matter (g/kg) | Nitrogen  (g/kg) | CEC (meq/kg) |
| --- | --- | --- | --- | --- | --- | --- | --- | --- | --- |
| 1 | Sept. 2018 | 23.4 | 55 | 18.3 | 6.2 | n.d | n.d | n.d | n.d |
|  | June 2019 | 14.1 | 52 | 33.8 | 6.1 | 47.5 | 82.3 | 3.21 | 186 |
|  | Aug. 2019 | 18.5 | 48 | 33.5 | 5.8 | 50.7 | 87.7 | 3.38 | 185 |
| 2 | Sept. 2018 | 26.2 | 44.7 | 26.4 | 6.7 | n.d | n.d | n.d | n.d |
|  | June 2019 | 20.9 | 47.9 | 31.3 | 5.2 | 29.2 | 50.5 | 2.45 | 119 |
|  | Aug. 2019 | 20 | 50.4 | 29.7 | 5.4 | 31.7 | 54.8 | 2.46 | 124 |
| 3 | Sept. 2018 | 16.1 | 33.4 | 48.5 | 6.3 | n.d | n.d | n.d | n.d |
|  | June 2019 | 13.3 | 48.7 | 38 | 5.7 | 42.8 | 74.1 | 2.89 | 141 |
|  | Aug. 2019 | 17 | 52.2 | 30.8 | 5.7 | 51.7 | 89.4 | 3.39 | 164 |


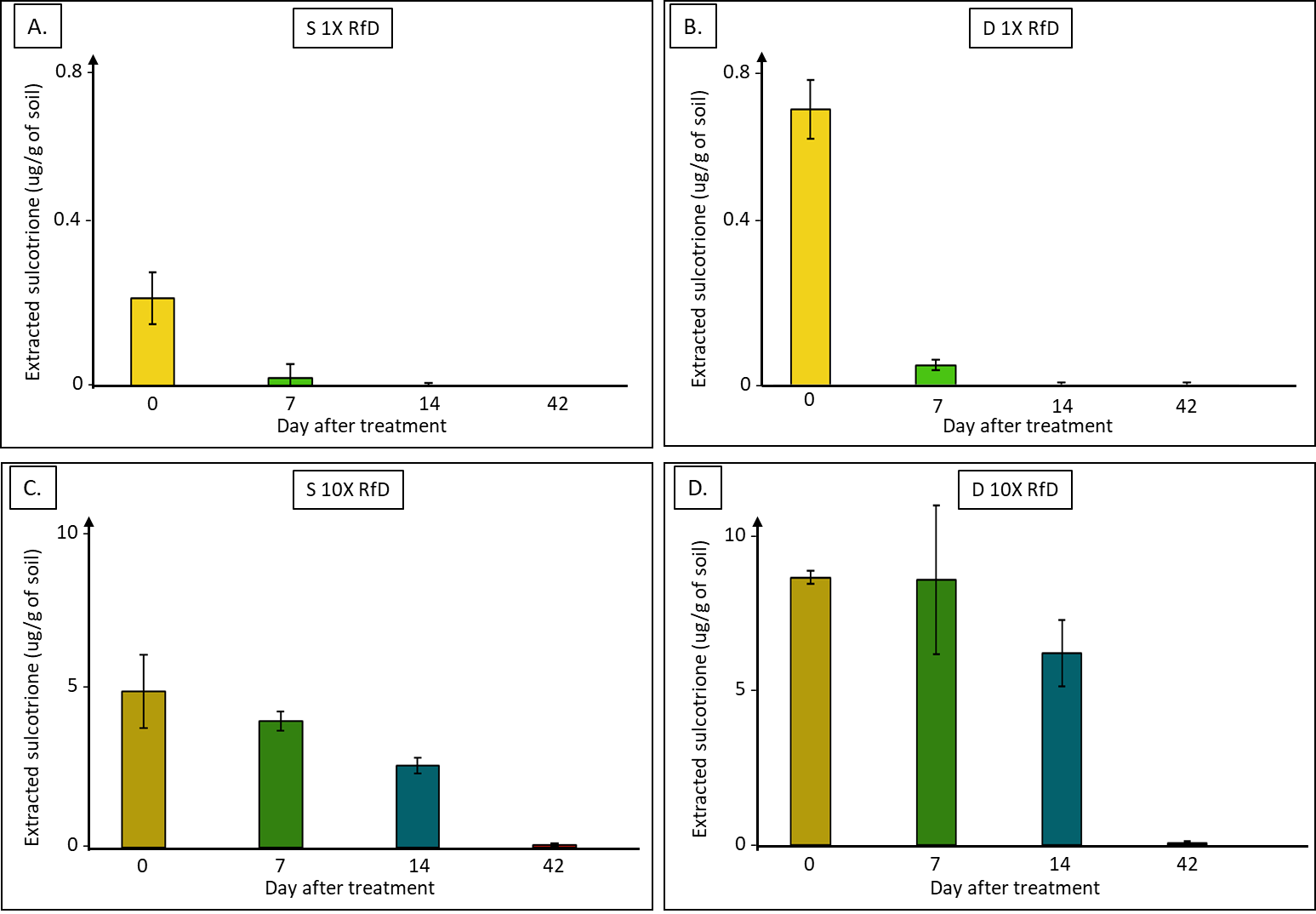


Figure S1. Dissipation kinetics of 1X RfD of sulcotrione **(A)** and Decano® **(B)** and 10X RfD of sulcotrione **(C)** and Decano® **(D)** in Perpignan soil microcosms. Standard deviations are indicated (n=3).

Table S2. Richness and diversity indices of the total bacterial community calculated for soil microcosms exposed to sulcotrione (S) and Decano® (D) applied at different concentrations (Control, 1X RfD, and 10X RfD) at 0, 7, 14 and 42 days. Mean values ± conﬁdence intervals are shown. Statistical analysis were led for each sampling day by comparing the different treatments to the control. Differences are indicated, if needed, by small letters (Kruskal–Wallis test P < 0.05).

| Day after treatment | Treatment | Observed species | PD whole tree | Simpson reciprocal |
| --- | --- | --- | --- | --- |
| 0 | S Control | 1367±61^b^ | 503±22^b^ | 206±25 |
|  | S 1X RfD | 1508±16^a^ | 544±9^a^ | 280±59 |
|  | S 10X RfD | 1520±35 ^a^ | 558±11^a^ | 282±48 |
|  | D Control | 2421±72 | 810±22 | 249±3 |
|  | D 1X RfD | 2237±187 | 760±44 | 208±76 |
|  | D 10X RfD | 2416±134 | 804±29 | 320±56 |
| 7 | S Control | 1270±46 | 482±12 | 91±27 |
|  | S 1X RfD | 1333±56 | 497±19 | 118±40 |
|  | S 10X RfD | 1326±88 | 499±12 | 115±30 |
|  | D Control | 2370±82 | 793±9 | 223±73 |
|  | D 1X RfD | 2283±300 | 768±74 | 270±109 |
|  | D 10X RfD | 2484±104 | 822±24 | 344±66 |
| 14 | S Control | 1485±31 | 558±11 | 174±3 |
|  | S 1X RfD | 1443±62 | 541±9 | 149±14 |
|  | S 10X RfD | 1474±51 | 520±19 | 165±57 |
|  | D Control | 2577±63 | 810±22 | 281±67 |
|  | D 1X RfD | 2447±66 | 760±44 | 205±105 |
|  | D 10X RfD | 2232±335 | 804±29 | 151±133 |
| 42 | S Control | 1540±16 | 536±9 | 241±75 |
|  | S 1X RfD | 1512±9 | 552±4 | 202±23 |
|  | S 10X RfD | 1548±24 | 543±6 | 218±41 |
|  | D Control | 2539±84 | 841±26 | 334±21 |
|  | D 1X RfD | 2596±41 | 848±17 | 353±18 |
|  | D 10X RfD | 2624±38 | 857±10 | 328±41 |


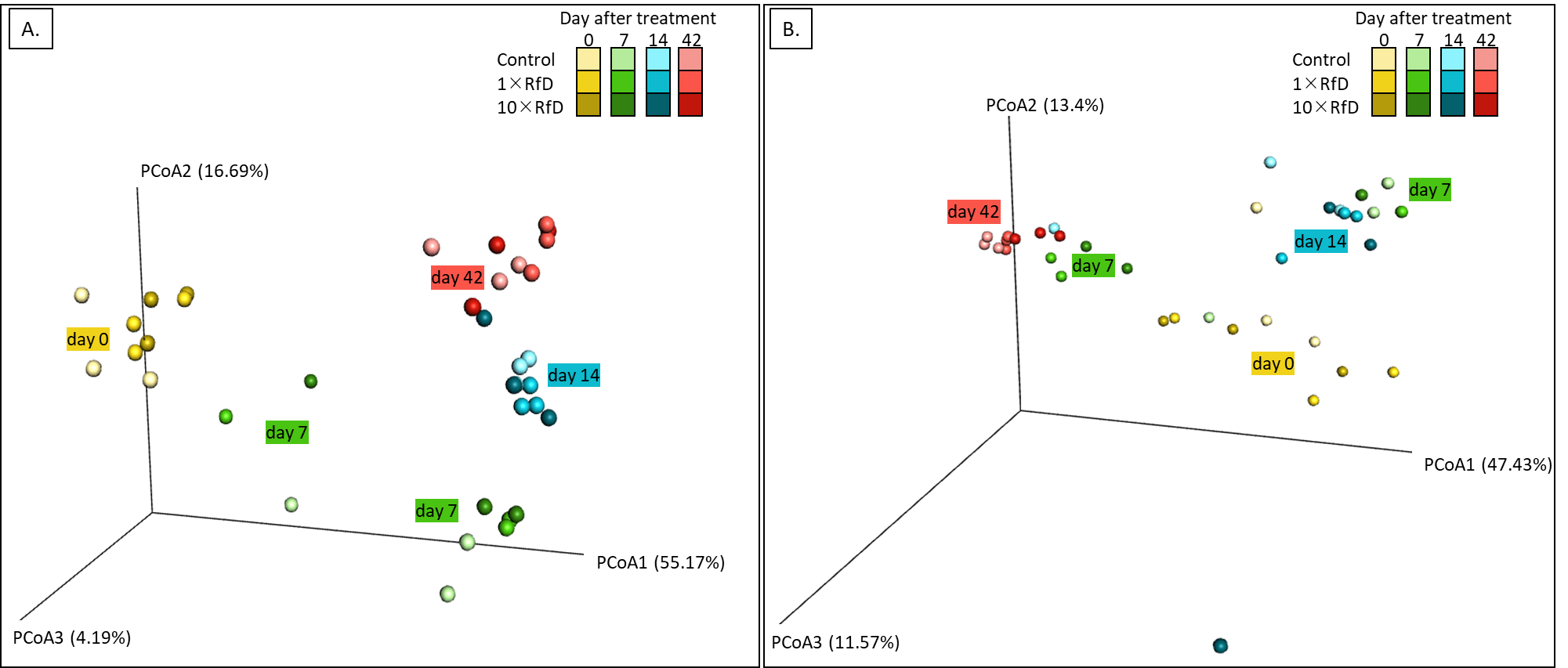


Figure S2. UniFrac analysis of the effect of sulcotrione **(A)** and Decano® **(B)** applied at different concentrations (Control, 1X RfD, and 10X RfD) on the total bacterial community composition of soil microcosms after 0, 7, 14 and 42 days of exposure. The ﬁrst three axes of the PCoA of the weighted UniFrac distance matrix of 16S rRNA amplicon Illumina sequencing are shown. The percent of variance explained by each axis is given.


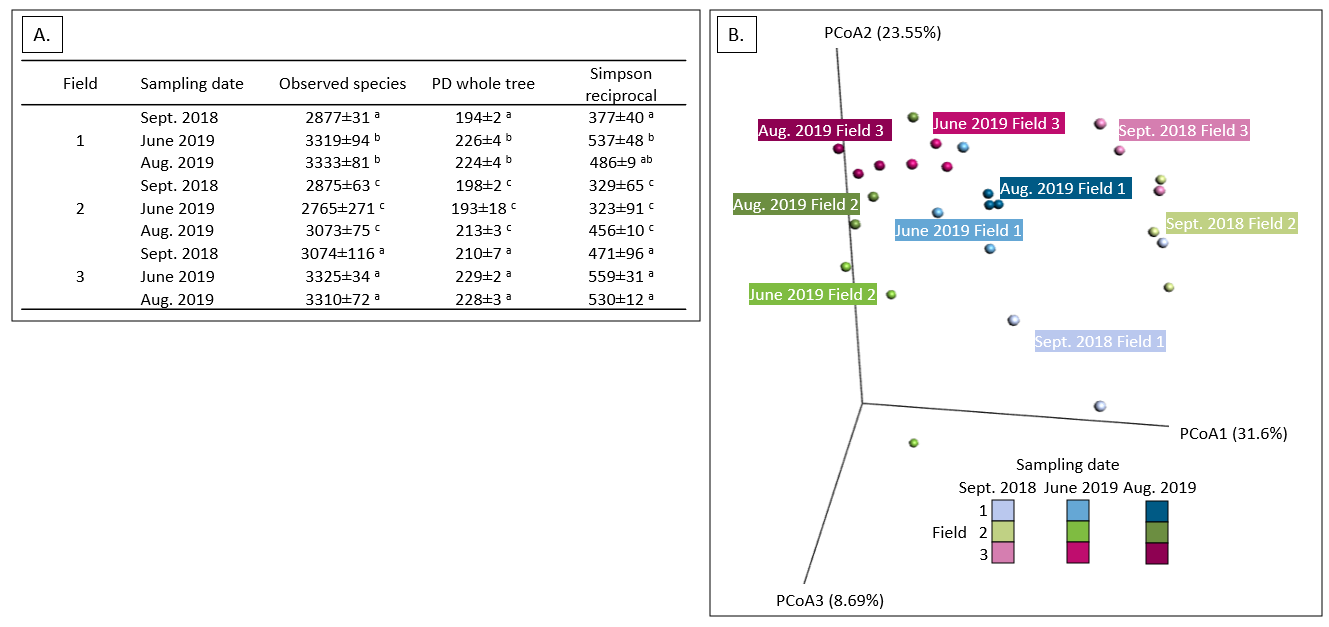


Figure S3. Analysis of the α-diversity **(A)** and the β-diversity **(B)** of the total bacterial community in soils of agricultural fields treated with PPPs (fields 2 and 3) or not (field 1) at different sampling times over a crop cycle (Sept. 2018, June 2019, Aug. 2019). **(A)** Richness and diversity indices of the total bacterial community (mean values ± conﬁdence intervals). ANOVA tests were conducted and p<0.05 was considered as significant and indicated by a small letter. **(B)** UniFrac analysis on the total bacterial community composition. The ﬁrst three axes of the PCoA of the weighted UniFrac distance matrix of *hppd* amplicon Illumina sequencing are shown. The percent of variance explained by each axis is given.
